# A robust data-driven approach for gene ontology annotation

**DOI:** 10.1093/database/bau113

**Published:** 2014-11-23

**Authors:** Yanpeng Li, Hong Yu

**Affiliations:** ^1^Department of Quantitative Health Sciences, University of Massachusetts Medical School, Worcester, MA, USA, ^2^Department of Computer Science, University of Massachusetts, Amherst, MA, USA and ^3^VA Central Western Massachusetts, Worcester, MA, USA

## Abstract

Gene ontology (GO) and GO annotation are important resources for biological information management and knowledge discovery, but the speed of manual annotation became a major bottleneck of database curation. BioCreative IV GO annotation task aims to evaluate the performance of system that automatically assigns GO terms to genes based on the narrative sentences in biomedical literature. This article presents our work in this task as well as the experimental results after the competition. For the evidence sentence extraction subtask, we built a binary classifier to identify evidence sentences using reference distance estimator (RDE), a recently proposed semi-supervised learning method that learns new features from around 10 million unlabeled sentences, achieving an F1 of 19.3% in exact match and 32.5% in relaxed match. In the post-submission experiment, we obtained 22.1% and 35.7% F1 performance by incorporating bigram features in RDE learning. In both development and test sets, RDE-based method achieved over 20% relative improvement on F1 and AUC performance against classical supervised learning methods, e.g. support vector machine and logistic regression. For the GO term prediction subtask, we developed an information retrieval-based method to retrieve the GO term most relevant to each evidence sentence using a ranking function that combined cosine similarity and the frequency of GO terms in documents, and a filtering method based on high-level GO classes. The best performance of our submitted runs was 7.8% F1 and 22.2% hierarchy F1. We found that the incorporation of frequency information and hierarchy filtering substantially improved the performance. In the post-submission evaluation, we obtained a 10.6% F1 using a simpler setting. Overall, the experimental analysis showed our approaches were robust in both the two tasks.

## Introduction

With the expansion of knowledge in biomedical domain, the curation of databases for biological entities such as genes, proteins, diseases and drugs, becomes increasingly important for information management and knowledge discovery. Ontology annotation, the semantic level of knowledge representation, plays a key role in the database construction. During the past decades, various ontology resources such as gene ontology (GO) (1) and medical subject headings (MeSH) (2), have been developed and shown great advantage to accelerate the process of biological and medical research. Among these resources GO has the largest number of concepts and records with an increasing demand of update rate, but the assignment of GO annotation of gene and gene products is a very time-consuming process because there are millions of gene names mentioned in biomedical literature, and the database curators (usually PhDs in biology) need to find evidence passages for each gene from over 20 million PubMed articles as well as assign one or more GO terms to each evidence passage from around 40 000 GO terms in the database (http://archive.geneontology.org/latest-termdb/go_daily-termdb.rdf-xml.gz). Therefore, GO annotation has become a major bottleneck in database curation workflows. Addressing the problem, during the past few years, researchers have attempted to use the techniques of information retrieval (IR) and machine learning for automatic GO annotation so as to accelerate the process. Benchmark data have been released for public evaluation since the BioCreative I 2004 GO Annotation Task (3), and TREC 2004 Genomics Track Triage Task and GO Annotation Task (4). In TREC Genomics Track 2004 (4), there were two tasks: the first task was to retrieve articles for GO annotation, where the best performance was 27.9% F-score and 65.1% normalized utility obtained by a logistic regression with bag-of-words and MeSH features; the second task was to classify each article into high-level GO classes: molecular function, biological process or cellular component, with the best F-score of 56.1% using a bag-of-words-based KNN classifier. These two tasks were both simplified version of GO annotation process, since they did not assign exact GO terms to certain gene. In BioCreative I challenge (3), the task was to assign GO terms to genes mentioned in text, exactly the same as the work of GO annotators. The evaluation was an IR-style pooling method that generated gold standard only from the predictions of the participants’ submitted results, and the evaluation measure was Precision rather than mean average precision (MAP) or recall, so that it was difficult to compare the overall performance of different systems. For example, some system achieved a precision of 34.2%, but only submitted 41 results, and some system achieved 5.75% precision with 661 predictions submitted (5). Nevertheless, based on the results it is no doubt that the task was rather difficult and the state-of-the-art performance was far from the requirement of practical use.

The GO task in BioCreative IV 2013 (6) was the most recent challenge evaluation for GO annotation which provided sentence-level annotated data and evaluation metric for both precision and recall. There were two subtasks: evidence passage extraction and GO term assignment, and both of them were evaluated by precision, recall and F1 measure, which was the first complete public evaluation study about the exact workflow of GO annotation. The best performance of the first task was 27% exact F1 and 38.7% relaxed F1; the best performance of the second task was 13.4% F1 and 33.8% hierarchy F1. Similar to BioCreative I, these tasks were still considered as extremely difficult ones with a large distance from database curators’ requirement. We think the difficulty lies in the following aspects: (i) text classification for 40 000 classes is much more difficult than binary classification task. It is even difficult for multiple human annotators to get the consistent annotation result. (ii) The training examples were not fully annotated. For example, in the first task there was no clear definition of a true negative example at sentence level (6). This means we only know for the given gene list which GO annotation is from which evidence sentence but are not sure if other sentences in the documents can also provide evidence or not for the same genes or other genes beyond the given list. (iii) A lot of annotations were based on sentence-level classification, which was a harder task than document classification because features from sentence were sparser and more difficult to model. (iv) The prior stages of GO annotation such as gene name recognition and normalization were also difficult tasks [around 80% state-of-the-art F1 score (7)]. Therefore, the errors from each step could be accumulated, thus producing a much worse overall performance than each individual step.

Since the entity recognition and normalization are classical tasks which have been worked on for many years, in this study we focus on the two new components introduced in BioCreative IV: evidence passage extraction (Subtask 1) and GO term prediction (Subtask 2), which can be separated from gene recognition/normalization for research. The first task can be viewed as the preliminary step of the second one, and they can all be treated as text classification for binary classes (Subtask 1) and for multiple classes (Subtask 2). Actually binary text classification has been one of the most extensively studied areas in IR, machine learning and natural language processing, e.g. the surveys (8)–(10). Therefore, on the one hand, a lot of previous works can be applied to this task, but on the other hand, it becomes more challenging to develop innovative approaches for further improvement. For text classification, it seems to be a common belief that bag-of-words features trained with supervised learning classifiers such as support vector machine (SVM) (11) and logistic regression (12) have created the state-of-the-art boundary and difficult to make big improvement if there are over thousands of training examples available. From the result of the critical challenge evaluations of text classification (4, 13, 14), it is difficult to find big improvement (e.g. over 10%) against supervised learning with bag-of-words features, although some domain specific methods, e.g. named entity features (15–17) were reported to achieve 1–2% improvement. Even though there might be big improvement for some specific dataset, it would be still challenging to develop a robust method for various datasets, just as if the appearance of Naïve Bayes, Logistic regression, SVM and KNN lead to revolutionary progress over the previous knowledge engineering-based approaches (8).

However, in the recent years, we noted that a novel strategy showed the potential of such big progress. Li *et al.* (18–20) developed a semi-supervised learning framework called feature coupling generalization (FCG) that learned new features from the co-occurrence of bag-of-words features in a large number of unlabeled data and found that it achieved over 5% improvement against the state-of-the-art bag-of-words features and elaborately designed lexical features in the challenging tasks of named entity recognition (7), relation extraction (21) and text classification (13). Recently, Li (22) proposed a more simplified approach called reference distance estimator (RDE) based on the FCG framework and gave a theoretical interpretation of why it worked. The theoretical analysis indicated that it improved performance because the method approximated a classifier trained with unlabeled-scale labeled data as if all the unlabeled data were correctly labeled. In the experiment of 10 text classification tasks, it was shown that RDE-based semi-supervised learning improved the AUC performance by over 10% against SVM, logistic regression and some other classical semi-supervised learning approaches; using 5000 labeled examples plus 13 million unlabeled ones the performance of the new method was close to the result of 13 million labeled examples. In this work, we explore if the approach can be used to enhance the performance of evidence sentence extraction, since in biomedical domain there are huge amount of unlabeled data available for RDE-based semi-supervised learning. This is the first application of RDE to benchmark challenging data, which is the major innovation of this work.

The second task addresses the final goal of GO annotation. The input data includes the evidence sentences and the gene IDs; the output is the GO terms assigned to each gene ID. It can be treated as the extension of the first task to many more classes, while the major difference is that the annotated sentences in BioCreative IV were not able to cover all the GO terms, so it is not straightforward to approach it as a classical text classification task due to the large number of out-of-vocabulary labels. In this case, one straightforward way is to use IR-based method which retrieves GO terms relevant to the certain query sentences, so that the GO terms out of the training data can also be assigned to the sentences as long as we can calculate the similarity between a sentence and a GO term. In this study, we tried various methods for query sentence construction, GO term representation, similarity function design, e.g. language model (23), and cosine similarity. During the experiment, we found that a small fraction of GO terms appeared frequently in documents and a large fraction of them appeared rarely. Therefore, similar to the idea of Page Rank, our retrieval model gave higher weight to the important (high-frequency) GO terms, and obtained big improvement on the F-measure. To our best knowledge, this strategy was firstly used in the GO task. In addition, to employ the annotated sentences to enhance the performance for the second task, we designed a classification task to predict high-level GO classes, since we found that a certain number of GO terms above the second level in GO concept hierarchy were included in the annotated sentences. We used the classification result to prune the result of IR so as to improve the precision of the system.

The rest of article is organized as follows: in Section 2 we describe the methodology for the two tasks. In Section 3, we present the experimental results. Finally, we give the conclusion and future work in Section 4.

## Methods

**Figure 1.**
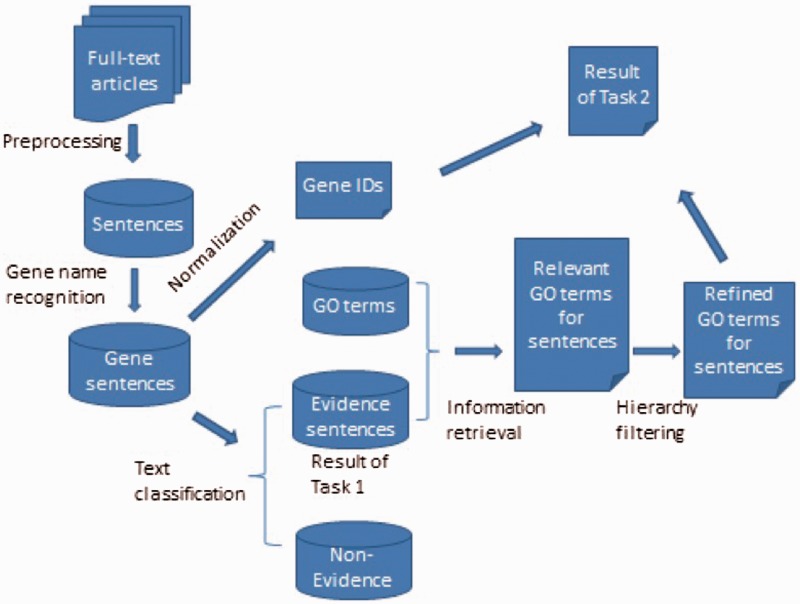
The framework of the GO annotation system.

In this section, we describe our methods for the two tasks evidence sentence extraction and GO term assignment respectively. Since the first task is part of the second task, the whole process can be described by the workflow in Figure 1, including the steps of preprocessing, named entity recognition/normalization, text classification, IR and hierarchy filtering. We first extracted sentences from full text articles, classified the sentence into evidence or non-evidence, and then used IR and filtering methods to get the final result of GO annotation. The submitted result for the first task was a list of evidence sentences together with Entrez Gene IDs mentioned in the sentences, and the result for second task was the predicted GO terms with associated gene IDs.

### Dataset and preprocessing

The annotated data provided in BioCreative IV (6) is a collection of full-text articles in PubMed Central: 100 ones for training, 50 for development and 50 for testing. The annotation was at the level of passage that was defined as either one or multiple sentences. The passages that indicated GO evidence were annotated as evidence passages, and for each evidence passage GO terms and associated Entrez Gene IDs were annotated. In the preprocessing stage, we split the sentences if the current token ended with a ‘.’, the next token was a whitespace and the next 2 token was not a lowercase letter. We removed all the sentences in the ‘References’ section, since no annotation was found in this section. For this task the generation of examples for machine learning was not straightforward. First, the flexible length of passage made it difficult to detect the boundary of passages. Second, the sentences were not fully labeled, that is, in the annotation guideline (6) there was no clear definition of a true negative example, so that noise would be introduced into both training and evaluation procedures. Since the sentence classification itself was already a difficult task, for simplicity we just considered each example as a sentence that contained at least one gene in the given list rather than merged sentences to generate passages.

### Gene name recognition and normalization

Gene named entity recognition and normalization are the important preprimary steps of biomedical text mining and have been studied for many years, and evaluated in many benchmark datasets, such as JNLPBA (24) and BioCreative challenges (7, 25, 26). The best F1 measures were less than 90% for the two tasks, respectively, which means the combination of the two steps tends to achieve an F1 under 80%, still a challenging problem. In the GO task, to make researchers focus on the text classification task only, the Entrez Gene IDs associated with each article were given in the training, development and test sets, and for test set the gene mentions exactly the same as those appearing in text were also given. Using this way the task organizers aimed to simplify the process of gene recognition and normalization to some extent, but systems were still required to develop these components, because: (i) for the non-evidence sentences, gene names and IDs were not annotated; (ii) the exact location of gene mention was not given, so we also needed to do entity recognition/normalization if we wanted to use gene information as features. For the training and development sets since the gene names provided were not exactly the same as the those in text, we applied a state-of-the-art gene named entity recognizer (19) with the best performance of 89.1% F1 on the BioCreative II Gene Mention dataset (7). Interestingly, this tagger was also developed based on the FCG semi-supervised feature learning strategy (18). After gene name recognition, we linked each recognized gene name to its Entrez Gene ID in the database, and we ignored the gene names with the IDs that could not be found the ID list of annotated article, since they were either not the focusing genes in the annotation data, or incorrect recognition results. For the test set, we just used a dictionary match for both named entity recognition and normalization because the gene information are exactly the same as those in the texts, although some of the names could not be found in the text in our experiment. After preprocessing and named entity recognition, we selected the sentences with the gene IDs in the annotated data as the positive (evidence) and negative (non-evidence) examples for the next text classification module.

### Text classification

The corpus statistics of the examples for text classification were listed in Table 1. There were 8285 labeled examples (965 + 665 + 4255 + 2400 in Table 1) in training and development data, which was not a small corpus comparing to the bench mark data, e.g. 20 news groups (http://qwone.com/∼jason/20Newsgroups/) and TREC Genomics Track (http://ir.ohsu.edu/genomics/). The positive examples were defined as evidence sentences in the gold standard; the negative ones were the sentences with the gene names but not annotated in the gold standard. As is discussed in the introduction, since the true negative sentences were not fully annotated in the gold standard, there could be noise in both training and evaluation. In the following we will present our approaches for feature and classifier design.

**Table 1.** Corpus statistics of the binary classification task

	Training data	Development data	Test
Number of positive examples	965	665	5494
Number of negative examples	4255	2400	

### Features

Since this task addresses the classification of sentences which are usually much shorter than paragraphs or the whole articles, the bag-of-words features from a local sentence tend to have high risk of data sparseness (18), which could result in a biased representation for low-frequency words and degrade the classification performance. Therefore, we tried to use two ways to enrich the representation: one was to use bag-of-words features from the context paragraph; another was to use RDE-based semi-supervised learning to learn high-level features from large unlabeled data. Totally we have eight types of features listed in Table 2, so that we can investigate the contribution of different strategies. As can be seen, the dimension of features was greatly reduced using RDE-based features, resulting in a semantic style representation. The detailed method for generating RDE features will be presented in the following section.

**Table 2.** The number of different types of features for the evidence sentence classification task

	Bag-of-words from sentence	Bag-of-words from sentence and paragraph	Bag-of-bigrams from sentence	Bag-of- bigrams from sentence and paragraph
Original lexical features	65 538	92 408	176 921	347 123
Features from RDEs	200	200	200	200

The first row is the corpus statistics from labeled data. The second row is the final feature set derived from the 200 RDEs.

### Classifier

As described in the introduction section, in this work we used RDE-based semi-supervised method (22) to learn new features from unlabeled data and investigate whether it could improve performance of evidence sentence classification. RDE is a simple linear classifier in the form of:

(1) $$f(x_{i},r)=\sum_{j}(P\left(r\text{|}j\right)-P(r))x_{ij}$$

where $x_{i}$ is the *i*th example represented by a Boolean vector of $x_{ij}$, *j* is the index of feature, and *r* is called a reference feature. The probability of P(r|j)−P(r) can be directly estimated from unlabeled data, as long as *r* is not the gold standard label. In the work (22), we showed in theory that if *r* is discriminative to the class label and highly independent with other features, the performance of RDE tends to be close to a classifier trained with infinite labeled data. The experiment on 10 text classification tasks showed that combining multiple RDEs from different reference features using only 50 00 labeled examples performed as well as a Naïve Bayes classifier trained with 13 million labeled examples in many tasks. Therefore, the application of RDE to the GO sentence classification can be straightforward, since it is also a text classification task.

In the previous work, we introduced a simple algorithm (22) that generated *k* RDEs from both labeled and unlabeled data and used the decision score of each RDE as the feature of a Logistic regression. The step processes of the semi-supervised algorithm are:

1. Rank candidate reference features by $\frac{1}{\left|I\left(r\right)\right|}\overset{}{\underset{j}{\sum }}P\left(j\right)(\left|\frac{P\left(j,r\right)}{P\left(j\right)\text{P}\left(r\right)}-1\right|)$ in ascending order and select top *k* reference features.
2. Construct *k* RDEs with the selected reference features in Step 1.
3. For each RDE remove the original features with $\left|\frac{P\left(j,r\right)}{P\left(j\right)\text{P}\left(r\right)}-1\right|>t$
4. Build a classifier using the decision score of each pruned RDE as a feature, and train the classifier with labeled examples.

**Figure 2.**
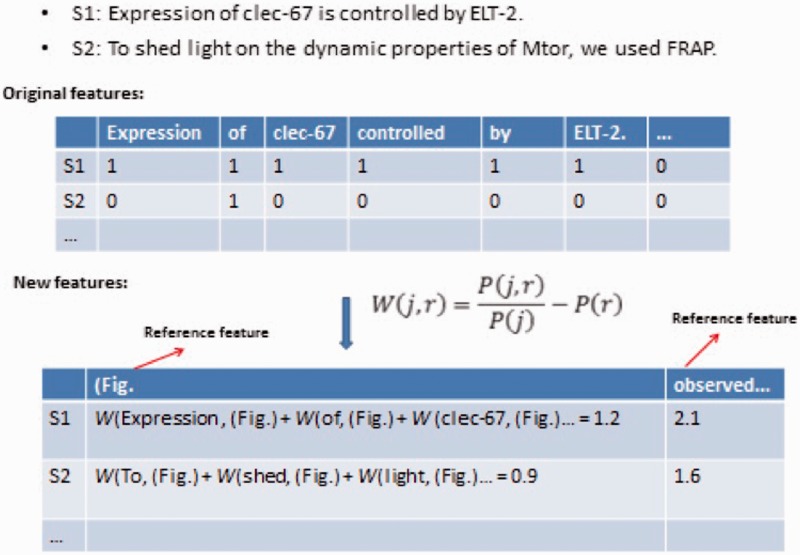
An example of RDE-based feature transformation for GO evidence sentence classification. S1 and S2 are two sentences. The example shows the part of original Boolean features, Reference features and new features generated by RDE semi-supervised learning.

Where $I\left(r\right)=\frac{P\left(r,\;y\right)-\alpha P\left(r,\bar{y}\right)}{P\left(r\right)}$, which is closely related to the precision of the reference feature *r* in distinguishing the class *y* from $\overset{�}{y}$. In Step 1, the other part $\overset{}{\underset{j}{\sum }}P\left(j\right)(\left|\frac{P\left(j,r\right)}{P\left(j\right)\text{P}\left(r\right)}-1\right|)$ reflects the expectation of the dependency of the reference features with other features, since the term $\left|\frac{P\left(j,r\right)}{P\left(j\right)\text{P}\left(r\right)}-1\right|$ measures the deviation of the mutual information between feature *j* and *r* against the fully independent case. The estimation of I(r) can be done by counting on labeled data and $\overset{}{\underset{j}{\sum }}P\left(j\right)(\left|\frac{P\left(j,r\right)}{P\left(j\right)\text{P}\left(r\right)}-1\right|)$ can be obtained from unlabeled data [see details in (22)]. This method encourages the selection of reference features with high precision in predicting the class *y* and independence with other features. In the GO evidence extraction task, e.g. the word features such as (‘Fig. and ‘observed’ are the top ranked reference features (Figure 2), since these features are good indicators of GO evidence sentences as well as relatively independent with other features. The algorithm converts original features to an enriched feature set with lower dimension (Figure 2). We found that the combination of these new features in a Logistic regression classifier achieved much better performance than original features. For clarity in the experiment, we defined SuRDE as the supervised RDE where the reference feature was the class label in the labeled training data, and SeRDE as the semi-supervised learning algorithm described above. These two methods were also compared in our previous work (22).

Rather than just applying the same algorithm, in this work we tried different methods for reference feature and the target classifier selection. The algorithm for reference feature selection in the work (22) was only based on the performance of individual RDE rather than the whole feature set. It is well known that in ensemble learning the combination of weak but complementary features could perform much better than the ensemble of strong but overlapping features. There is still no theory to suggest the optimal reference feature selection for the ensemble method. Also logistic regression may not be the optimal one for combining different RDEs. Therefore, in the experiment, we compared different strategies for reference feature selection, e.g. Chi-square, and frequency-based ranking, and different classifiers for RDE ensemble such as SVM and random forest. We also combined classifiers with different features to make further improvement.

Our unlabeled data included around 10 million sentences in a subset of full text articles from the journal Science, Nature, PNAS, PLOS Genetics, Genome Research, RNA and NAR. These full texts were downloaded under the license of the library of University of Massachusetts Medical School. Since we only sampled the ‘gene sentences’ in labeled data, to be consistent for the unlabeled data we also used the gene mention recognizer (19) to get the 10 million sentences that contained gene names. Since the training of RDE can be done by counting (22), it is very efficient to work on terabyte-scale unlabeled data. To our best knowledge, there are very few semi-supervised learning methods that can handle such scale of unlabeled data (27).

After text classification, we got predicted evidence sentences. In order to generate the result for Subtask 1, the following step was to link the evidence sentences to the candidate gene IDs, since the official evaluation required specific gene for a given evidence sentences. We used a straightforward method: if the gene appeared in the evidence sentence, the sentence and gene ID would be submitted as the final result. Actually, there could be some error during the linking process (See also the section of error analysis). For example, although a gene appears a sentence, it could not be the focusing gene in that sentence. In this work, we did not consider that case in our method, but we think it will be an interesting topic for future study.

### Information retrieval

The IR-based method aimed to search the candidate relevant GO terms for a certain sentence. In the method, each positive sentence in Subtask 1 was treated as a query, and the GO term most relevant to the sentence was returned as the candidate predictions. The task is a little different from traditional document retrieval, since the GO terms are usually much shorter than a document, and there is concept hierarchy relationship between the GO terms. We tried to use different ways to represent GO terms including the words in the term, the synonyms and the narrative sentences in the description. We also tried to used Indri (23), a state-of-the-art IR toolkit of language model. However, we found that the simplest method based on cosine similarity worked best for this task. Therefore, this simple approach was employed in the submitted runs and the following experiments.

**Figure 3.**
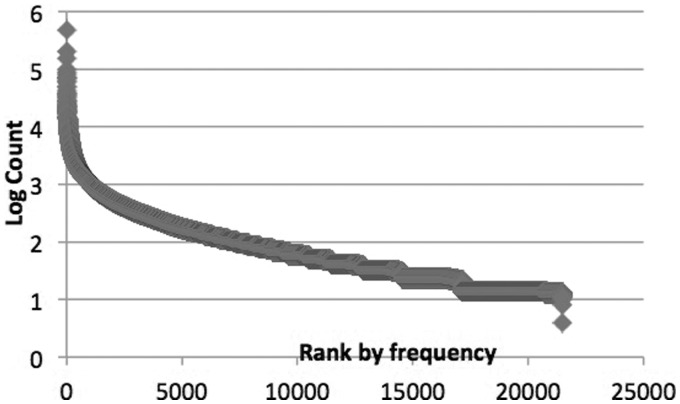
Distribution of GO terms appearing in biomedical literature.

Furthermore, in the experiment, we found the frequency of GO terms had a big impact on the performance of ranking, since the occurrence of GO term in documents followed a power law distribution, where a small fraction of GO terms appeared frequently in a lot of documents, and most GO terms appeared rarely (Figure 3). Therefore, if we give higher weight to the important GO terms (high-frequency terms), the F-score tend to be much better, just similar to the idea of Page Rank algorithm in Web search, which prefers the important pages linked by a lot of other pages. Our ranking function is:

(2) GORank(sentence, GO term) =#of Common words in sentence and GO term#of words in sentence#of words in GO termlog(count(GO term))

where the first part is the cosine similarity of the sentence and GO term, and count(GO term) is the number of documents related to the GO term in the Gene Ontology Annotation (GOA) databases (http://www.geneontology.org/GO.downloads.annotations.shtml). In the GORank function, both lexical similarity and frequency of GO terms are considered. In the experiment, all the words were lowercased, since we found it worked a little better. The current format of the GORank function was obtained through many experiments where we found this type of combination performed better than the weighted linear combination or the formula with unlogged counts.

### Hierarchy filtering

Using the fully unsupervised manner, we were able to get a ranked list of GO terms for each sentence, but the annotated sentences were not employed. One of the major motivations of the challenge to investigate how much the annotated data can help to improve the performance of GO annotation. In order to make use of the information in the annotated sentences to improve the performance, after the ranking, we built a classifier for 12 high-level GO classes trained on labeled sentences to prune the result. Since there are around 40 000 GO terms in the GO database and only around 500 terms in the training data, it is difficult to build a classifier for the whole vocabulary of GO terms, but it is much easier to build a classifier for high-level GO terms, since the vocabulary becomes much smaller when moving to the root of the Ontology concept tree. According to the database (http://archive.geneontol ogy.org/latest-termdb/go_daily-termdb.rdf-xml.gz), there are three GO terms (i.e. Cellular component, Biological process and Molecular function) in the first level, and 60 terms in the second level, of which 11 most frequent terms in training data were used to build 12 binary classifiers (one for ‘other’ class) to assign the most relevant terms. We define a filtering threshold *n* as the number of *n* most relevant high-level GO classes to the sentence determined by the classifiers. If the highest ranked GO term by GORank is in the *n* classes, it will be selected as a positive result. In the contest, supervised RDE was used, since we did not have time to test the semi-supervised method before submission. In the future, we will consider the application of semi-supervised RDE in both hierarchy filtering and classification for high-frequency GO terms, e.g. the top 2000 terms in the global annotation.

## Results

In this section, we present the experimental results for the two subtasks, respectively. For Subtask 1, we investigate the performance of different features and classifiers, the impact of reference feature selection and unlabeled data. For Subtask 2, we compared the performance of different query and GO term representation methods, retrieval models and methods for hierarchy filtering. Table 3 describes the methods used in our submitted runs.

**Table 3.** Method description of submitted runs

Subtask	Run ID	Method description
1	Run 1	RDE, 110 reference features, Logistic Regression, classification threshold = 0.16
1	Run 2	RDE, 110 reference features, Logistic Regression, classification threshold = 0.18
1	Run 3	RDE, 110 reference features, Logistic Regression, classification threshold = 0.14
2	Run 1	GO Rank, Hierarchy filtering, GO terms with the count over 2000 in the GOA database for ranking. classification threshold (Subtask1) = 0, filtering threshold = 6
2	Run 2	GO Rank, Hierarchy filtering, GO terms with the count over 500 in the GOA database for ranking, classification threshold (Subtask1) = 0, filtering threshold = 8
2	Run 3	GO Rank, Hierarchy filtering, GO terms with the count over 2000 in the GOA database for ranking, classification threshold (Subtask1) = 0.16, filtering threshold = 2

In the table, ‘classification threshold’ is the threshold of the Logistic regression classifier with 110 RDE features. The ‘filtering threshold’ is the number of *n* most relevant high-level GO classes to the sentence determined by the classifiers. If the highest ranked GO term by GORank is in the *n* classes, it will be selected as a positive result.

### Results for Subtask 1

Table 4 shows the performance of different methods on the test set using the official evaluation measures. The baseline was a simple rule-based method that treated all the gene sentences as evidence sentences, achieving the highest recall but lowest precision. However, since the relaxed recall is only 65% but not 100%, it can be inferred that the named entity recognition and normalization tasks accounted for a significant proportion of errors. Using different classifiers trained on the annotated corpus, Precision and F1 improved while recall decreased. It is promising to see that all the runs based on RDE achieved better F1 than SVM (11) and logistic regression (12), and the incorporation of RDE produced significant improvement on F1comparing the performance of Logistic regression (F1 17.4% and 28.6%) with the best run with RDE (F1 22.1% and 35.7%), which justified the success of the application of RDE to this task. The combination (Result 11) of RDEs with unigram features (Result 8), bigram features (Result 9) and supervised logistic regression (Result 3) improved the performance against the best individual ones, indicating the semi-supervised framework was able to incorporate rich feature set to enhance the performance. Note that for Result 11 in Table 4 we just used the mean of the decision scores of the individual classifiers as the combination score, so there was little risk of overfitting caused by classifier ensemble. The classification thresholds of all the classifiers were tuned based on the F1 measures on the development set, so at this level the comparison was fair. The reason for the better performance here than the submitted runs is that we used more reference features (200 vs. 110 in submission), the incorporation of bigram features and the combination method.

**Table 4.** Comparison of different methods on test set of Subtask 1

ID	Method	Precision (exact) (%)	Recall (exact) (%)	F1 (exact) (%)	Precision (relaxed) (%)	Recall (relaxed) (%)	F1(relaxed) (%)
1	NER, no classifier (baseline)	9	**39**	14.7	15.2	**65.5**	24.6
2	SVM (words)	11.1	36.3	17	18.4	60.3	28.2
3	Logistic (words)	11.8	33	17.4	19.4	54.3	28.6
4	SuRDE (words)	12.8	32.6	18.4	20.4	51.9	29.3
5	SeRDE (Run 1)	14.6	28.6	19.3	23.9	46.9	31.7
6	SeRDE (Run 2, our best submission)	15.3	25.9	19.3 ( + 31.3%)	25.8	43.7	32.5 (+32.1%)
7	SeRDE (Run 3)	14	31.1	19.3	22.6	50.3	31.2
8	SeRDE (200 refs, words)	16.7	24.5	19.9	27.7	40.6	32.9
9	SeRDE (200 refs, bigrams)	17.1	23.6	19.8	27.5	38	31.9
10	8+9	18.3	24.3	20.9	29.8	39.7	34.1
11	3+8+9	**18.6**	27	**22.1 (+50.3%)**	**30.1**	43.7	**35.7 (+45.1%)**

‘NER, no classifier’ is the method that uses all the gene sentences as evidence sentences. SuRDE and SeRDE are the supervised and semi-supervised RDEs defined in (22). All the classifiers were trained with the labeled examples in training and development sets in Table 1. Logistic regression was used to integrate RDE features from Method 5 to 8. Random forest was used in Method 9. The ensemble Method 10 (8+9) used the mean of the decision scores of the individual classifiers (Methods 8 and 9) as the combination score. Method 11 was the combination of Methods 3, 8 and 9 in the same way.

Since the evaluation takes into account many other factors such as gene normalization and gene-sentence linking, we cannot see clearly the performance of the text classification task itself in Table 4. Therefore, we showed the result of the binary classification task in Table 5, where it is clear to see the improvement of RDE against the other machine learning approaches. The significant improvement in AUC indicates a more robust result than F1, since AUC is insensitive to the threshold selection. The comparison of the performance in Tables 4 and 5, reveals that due to the introduction of more training data, there is bigger improvement on the test set for supervised classifiers in both F1 measures, in particular for SVM and Logistic regression, while Semi-supervised RDE showed much more robust performance on the two different sets.

**Table 5.** Comparison of different methods on development set of Subtask 1

	F1 (binary) (%)	AUC (binary) (%)	F1 (exact) (%)	F1 (relaxed) (%)
NER, no classifier	-	-	14.6	22.8
SVM (baseline)	38.4	62	14.9	23.4
Logistic	36	61	15.4	23.7
SuRDE	45.2	71	17.9	27.4
SeRDE (200 refs, words)	49.2	74.6	18.7	29.6
SeRDE (200 refs, bigrams)	48.8	74.2	18.5	29.7
SeRDE (200 refs, words + bigrams)	50.2	76.5	19.2	30.7

F1 (exact) and F1 (relaxed) are the official evaluation measures. The F1 (binary) and AUC (binary) are the performance on the binary sentence classification task defined in ‘Method’ and Table 1.

In Table 6, we compared the performance of different classifiers for the RDE-based features. In our experiment, we found that logistic regression and Random Forest were the two of the best classifiers for the RDE features. Logistic regression achieved the best F1 score for unigram features and Random Forest achieved better overall performance for bigram features. In the previous work (18), we also found similar cases where the new features obtained by feature co-occurrence worked better with non-linear classifier such as SVM with RBF kernel, since they have much lower dimension (e.g. 200 for all the runs in Table 6 feature space just like a semantic level representation. Here we found that the RDE features with Random forest showed better accuracy and efficiency than other non-linear classifier such as SVM with non-linear kernel. Random Forest (28) is one of state-of-the-art non-linear classifiers which utilizes repeatedly random feature discretization and conjunction to generate high-order discriminative and diverse features for learning. On the one hand, it is encouraging to see the good results obtained by RDE, especially for the big improvement on bigram features using Random Forest, which shed light on the methodology to exploit high-order features which were not utilized well in classical methods for IR and NLP due to data sparseness. On the other hand, we see the potential for further improvement that there is still the gap between the current bigram result and its upper bound, e.g. the semi-perfect classifier defined in the work (22), since the performance of bigram is still lower than unigram but intuitively bigram should perform better.

**Table 6.** Comparison of different features and classifiers on test set

Classifier for RDE features	Original features	F1 (exact) (%)	F1 (relaxed) (%)
Logistic	Sentence, words	18.8	31.1
Random Forest	Sentence, words	19.3	32.6
Logistic	Sentence, bigrams	19.2	31
Random Forest	Sentence, bigrams	19.5	32.8
Logistic	Sentence + Paragraph, words	**19.9**	**32.9**
Random Forest	Sentence + Paragraph, words	19.4	32.4
Logistic	Sentence + Paragraph, bigrams	19.6	30.6
Random Forest	Sentence + Paragraph, bigrams	19.8	31.9

**Figure 4.**
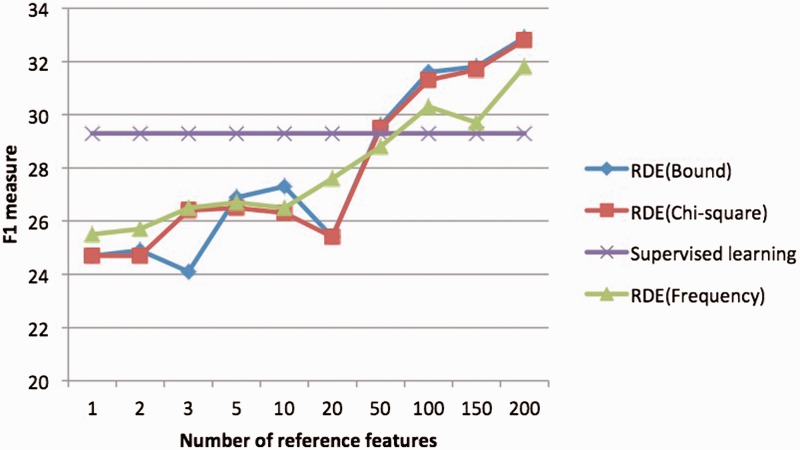
the relation between the number of reference features and F1 on Subtask 1.Only the unigram word features were considered in the experiment the classifier for RDE features is Logistic regression.

In Figure 4, we analyzed the impact of reference feature selection. We compared different strategies for reference feature selection including the metric obtained by theory (22), and the chi-square method used in previous wok (18), and the most naïve method—the frequency-based method which just selects top 200 high-frequent features as the reference features. In the experiment, we found the frequency of the reference features was a very important factor to achieve good performance, and were surprised to see that frequency-based reference features worked almost as well as other supervised methods. We have the following reasons for that: (i) independence is a very important factor in reference selection (22) and high-frequent words tend to be independent with all the other words. (ii) The joint probability of high frequency features can be estimated more accurately than low frequency features, even given a large amount of unlabeled data. Also according to our analysis there were very few highly indicative word features in this task, so the labeled data could not help much to the reference feature selection. It is also promising to see from Figure 4 the ensemble of reference features improved the performance increasingly with more reference features incorporated.

**Figure 5.**
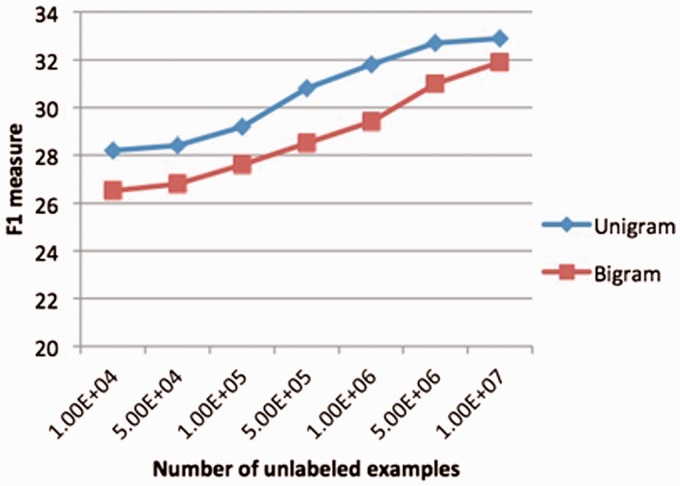
Performance varied with number of unlabeled data. The reference features are the bound-based reference features in section 2.3.2 and Figure 4. classifiers for RDE features are Logistic Regression (for unigrams) and random forest (for bigrams).

We also observed the impact of the scale of the unlabeled data for this task (Figure 5). It is interesting to see these unlabeled data becomes a valuable resource for machine learning and the RDE-based semi-supervised learning scaled well for the big data. We believe it will play a very important role in the future for big data mining due to the great scalability.

### Results for Subtask 2

Tables 7 and 8 show the performance of various methods on the test and development data in Subtask 2. As can be seen, cosine similarity performs much better than Indri, a classical language model-based method, on exact performance but inferior on hierarchy performance. The incorporation of definition for GO term representation decreases almost all the performance. The possible reason for these different results from traditional IR task is that the Indri could work well for the document-level retrieval but for the much shorter documents and representation based on narrative description will introduce noise for the retrieval. It is interesting to see that GORank outperformed both cosine similarity and Indri on most of the performance measures. Methods that incorporate the frequency of GO terms (i.e. frequency-based filtering and GORank) achieve significant improvement. Run 3 achieved the best performance on exact precision and F-score on the test set. Hierarchy filtering improved the precision and F-score in both development data and test data. The simple method that used GORank and hierarchy filtering achieved the best overall performance on test set, but not the best on development set, so this run was not submitted for the official evaluation.

**Table 7.** Performance of different methods on the test set of Subtask 2

Method	Precision (exact)	Recall (exact)	F1 (exact)	Precision (hierarchy)	Recall (hierarchy)	F1 (hierarchy)
Indri (baseline)	1%	3%	1.5%	9.9%	33.1%	15.2%
Indri + definition	0.8%	3%	1.3%	8.5%	34.7%	13.7%
Cosine	2.4%	7.6%	3.6%	7.2%	**40.6%**	12.2%
GORank	5.9%	**14.3%**	8.4%	13.5%	31.8%	19%
GORank + hierarchy	**10.6%**	10.6%	**10.6% (+606.7%)**	21.6%	21.2%	21.4%
Cosine + Frequency	4.6%	9.8%	6.2%	15.1%	28.4%	19.7%
GORank + frequency	5.5%	10.7%	7.3%	17.4%	27.5%	21.3%
GORank + frequency + hierarchy (Run 3)	9.5%	6.7%	7.8%	**27.8%**	16.1%	20.4%
GORank + frequency + hierarchy (Run 1)	5.2%	11.2%	7.1%	17%	32%	**22.2% (+46%)**
GORank + frequency + hierarchy (Run 2)	4.9%	14.3%	7.3	12.7%	36.8%	18.8%

‘Indri’ is a language model-based method (23). ‘Definition’ means appending the definition of GO terms to expand the text representation. ‘Cosine’ is the similarity function in the first part of Formula (2). ‘Frequency’ is to limit GO vocabulary to the high-frequency GO terms (Table 3). ‘Hierarchy’ is the high-level GO class-based filtering.

**Table 8.** Performance of different methods on the development set of Subtask 2

Method	F1 (exact)	F1 (hierarchy)
Indri (baseline)	1.3%	11.8%
GORank	5.9%	**17.3% (+46.6%)**
GORank + hierarchy	6.6%	16%
GORank + frequency + hierarchy (Run 3)	5.9%	12.7%
GORank + frequency + hierarchy (Run 1)	6.9%	16.3%
GORank + frequency + Hierarchy (Run 2)	**6.9% (+430.8%)**	16.4%

### Error analysis

Since there are multiple components in the GO annotation system (Figure 1), it is important to know the error distribution in each step. In the error analysis stage, we investigated the distribution of error types in different steps via observing the performance change after incorporation of gold standard. For some steps, e.g. named entity recognition, evidence extraction, we replaced the predicted result by the gold standard to investigate the impact of each step. In Table 9, the first row is the baseline with the best performance in the two subtasks. When the gold standard gene sentences were mixed with the candidate sentence set, there is around 10% absolute improvement for Subtask 1, indicating that the impact of entity recognition and normalization is at least 10%. Note that our system first identified sentences with gene names (*S*) and then classified them into positives (*Sp*) and negatives (*Sn*). The first row in Table 9 was not to replace all the gene sentences (*S*) by the gold standard sentences (*G*) but to ‘Add the gold standard evidence sentences (*G*) to the gene sentences (*S*) to be classified’. Therefore, the final merged set (*S+G*) includes all the negative instances (*Sn*), which accounted for the 46% relaxed F1 rather than 100% in Table 9. Since the set *G* can be viewed as part of gold standard for gene name recognition/normalization, the method actually added some gold standard to the gene sentences recognized by the system. It was difficult to know the exact impact of entity recognition and normalization, since there was no complete entity annotation in BioCreative IV GO corpus. Another 7% improvement on relaxed F1in Subtask 1 was obtained by replacing gene IDs by the same IDs in the evaluation, since some errors occurred when linking the gene IDs and evidence sentences. For example, the mention of gene in the sentence does not necessarily mean the sentence describes the evidence of this gene. The Method 4 in Table 9 used the gold standard result of Subtask 1 as the input of Subtask 2, and yielded around 10% absolute improvement on Subtask 2. In the last analysis method, we can see that if the high frequency GO terms are correctly predicted, the micro F1 for Subtask 2 can be greatly improved to over 60%. This result also supports our attempt of incorporating frequency information into the IR model to enhance GO annotation. From the analysis, we can conclude the large proportion of error lied in the classification for the most frequent classes, e.g. around 500 ones with the frequency higher than 2000, and gene named entity recognition/normalization. Therefore, these two steps should be our research focus in the future.

**Table 9.** Performance analysis via incorporation of gold standard in different steps

	Analysis method	F1 (exact, Task 1) (%)	F1 (relaxed, Task 1) (%)	F1 (exact, Task 2) (%)	F1 (hierarchy, Task 2) (%)
1	Baseline, Result 11 in Table 3 (Subtask 1), Result 5 in Table 6 (Subtask 2)	22.1	35.7	10.6	21.4
2	Add the gold standard evidence sentences to the gene sentences to be classified	31.8	46.2	12.5	24.9
3	Based on Result 2, replace all the gene IDs by the same ID for Subtask1	36	53.4	12.5	24.9
4	Use the gold standard of Subtask 1 as the input of Subtask 2	100	100	19.6	33.1
5	Replace the final result by the gold standard of Subtask 2 only for the terms with the frequency over 2000 in GO annotation databases	100	100	61.2	65.4

## Conclusion

We present the application of RDE-based semi-supervised learning to the first subtask, and GORank with RDE-based filtering for the second subtask. Our novel methods lead to big improvement on F1 measure and robustness against the classical text classification and IR methods on the two subtasks. For the first task, it is very promising to see that over 20% improvement introduced by reference distance learning from unlabeled data, which indicates the great potential for the next revolutionary progress in text classification, natural language processing and IR. The most encouraging thing is that the high-order features, e.g. bigrams can be utilized well to achieve good performance, and we believe there is great potential for exploiting more types of high-order word features, since data sparseness, the major barrier of using high order features can be overcome by RDE to a certain extent. In the future we will continue to develop machine learning methods as well applications based on this idea.

The second subtask seems more difficult because of the large vocabulary of classes. There are also similar problems in the image annotation where the performance of thousands of classes tends to be much lower than the binary classification. We think that no matter using text classification or IR technique, the representation of text and GO terms plays a central part. We will try to apply RDE-based semi-supervised learning to this task since it learns representation towards the optimal one in theory. For the labels not in the annotated data, if we are able to find some good (accurate and independent) reference features using external resources, we may achieve equally well performance as supervise learning. The challenge lies in collecting fully or semi-annotated data for all the 40k classes, since the proportion of rare classes in the GO vocabulary is large (Figure 3), and the number of annotated sentences is limited. However, one good news is that due to the power law class distribution (Figure 3), the big class vocabulary would not hurt much the micro level evaluation metric, e.g. the F-score used in official evaluation. Therefore, the classification for the minority of classes (e.g. around 500 high-frequency classes in the experiment) can be viewed as a goal not very far from the final goal (classification for 40k classes). It is much more efficient to try various supervised or semi-supervised methods to improve the performance on the 500 classes than 40k classes. It is reasonable that if we want to get big improvement on the micro F1 measure, we must solve the classification problem for high-frequency classes first as an important preliminary step. One simple specific way to do this is to build a supervised classifier for the small amount of high frequency classes (e.g. 500 classes) and then use IR method for the rest of classes (e.g. 38 000 GO terms). Another potential aspect for further improvement is the incorporation of the information of certain genes, since the gene information is the heart of the GO annotation and various types of gene information in the databases, e.g. existing annotation or genotype data can be used as additional features for machine learning.

## Funding

National Institutes of Health (GM095476). Funding open access charge. National Institutes of Health (GM095476).

*Conflict of interest*. None declared.
